# Ten-year retrospective data analysis reveals frequent respiratory co-infections in hospitalized patients in Augsburg

**DOI:** 10.1016/j.isci.2024.110136

**Published:** 2024-05-28

**Authors:** Martin Krammer, Reinhard Hoffmann, Hans-Georg Ruf, Avidan U. Neumann, Claudia Traidl-Hoffmann, Mehmet Goekkaya, Stefanie Gilles

**Affiliations:** 1 Environmental Medicine, Faculty of Medicine, University of Augsburg, Augsburg, Germany; 2 Institute for Medical Information Processing, Biometry, and Epidemiology - IBE, LMU Munich, Munich, Germany; 3 Pettenkofer School of Public Health, Munich, Germany; 4 Institute for Laboratory Medicine and Microbiology, University Hospital of Augsburg, Augsburg, Germany; 5 Institute of Environmental Medicine, Helmholtz Center Munich, German Research Center for Environmental Health, Neuherberg, Germany; 6 Christine-Kühne-Center for Allergy Research & Education (CK-Care), Davos, Switzerland

**Keywords:** Microbiology, Virology

## Abstract

Clinical data on the types of respiratory pathogens which are most frequently engaged in respiratory co-infections of children and adults are lacking. We analyzed 10 years of data on a total of over 15,000 tests for 16 viral and bacterial pathogens detected in clinical samples at the University Hospital of Augsburg, Germany. Co-infection frequencies and their seasonal patterns were examined using a proportional distribution model. Co-infections were detected in 7.3% of samples, with a higher incidence in children and males. The incidence of interbacterial and interviral co-infections was higher than expected, whereas bacterial-viral co-infections were less frequent. *H. influenzae*, *S. pneumoniae*, rhinovirus, and respiratory syncytial virus (RSV) were most frequently involved. Most co-infections occurred in winter, but distinct summer peaks were also observed, which occurred even in children, albeit less pronounced than in adults. Seasonality of respiratory (co-)infections decreased with age. Our results suggest to adjust existing testing strategies during high-incidence periods.

## Highlights

• Respiratory co-infections (bacterial and viral) studied in a large clinical dataset
• Higher frequency of co-infections in children and males, peak in winter
• Overall frequency was 7.3%; interviral and interbacterial co-infections dominated
• A distinct summer peak was observed in adults’ and in children’s samples

## Graphical abstract

**Figure:**
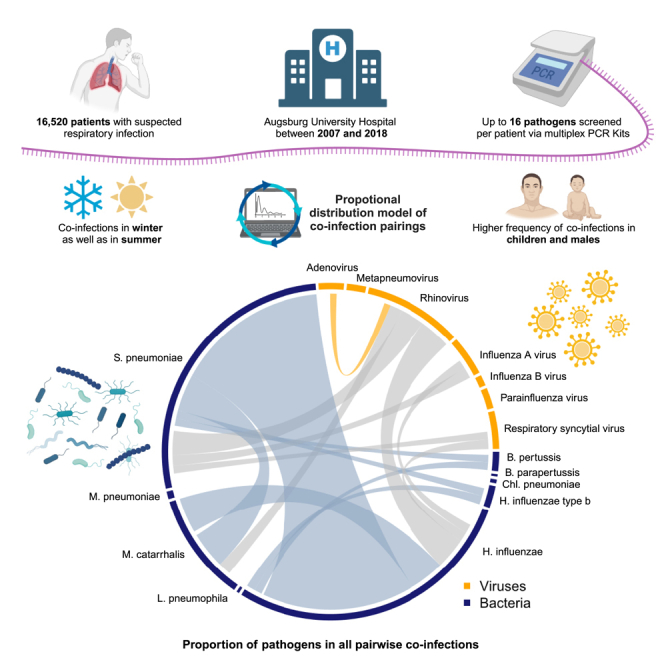

## Introduction

In the wake of the COVID-19 pandemic and its enormous global burden of disease, research on concurrent respiratory infections with more than one pathogen (so-called co-infections^1^) has also received an increased impetus. Co-infections have been found to occur in approximately 5.0%–62.0%^2^^,^^3^ of patients with respiratory tract infections; the frequencies found depended on investigated age groups, disease severity, infection location, pathogen focus, methods of pathogen detection, number of pathogens tested for, underlying data period or environmental circumstances, and other parameters.

The current state of research suggests that many pathogen pairings do not occur incidentally and that direct and indirect interaction effects are involved.^4^^,^^5^^,^^6^ Physiologic mechanisms (e.g., epithelial damage, enhancement of pathogen adhesion, and alteration of airway function), immunologic processes (in particular, the reduction in the number and functions of innate and adaptive cells), as well as direct pathogen interactions were identified as major pathways of pathogen interference and are potentially decisive for multi-microbial pathogenicity.^1^ A growing body of evidence further points to the adverse impact of co-infections on clinical outcomes, particularly increased hospital admission, length of stay, patient complexity, mortality, and cost of care,^3^^,^^7^^,^^8^^,^^9^^,^^10^ although inconclusive results have been reported for interviral co-infections.^2^^,^^11^ A better understanding of co-infections is thus of high prognostic and therapeutic relevance, e.g., through adapting testing strategies, initiating targeted therapies earlier, and avoiding unnecessary treatments.^10^

Novel models for the assessment of pathogen co-occurrence and interference allow to draw an increasingly profound picture on co-infection events.^4^^,^^5^ Nevertheless, evidence on co-infections still needs to be fully transferable to various regional contexts, is commonly limited to specific therapeutic situations, and/or focuses on a single pathogen (type) and its co-infecting agents. Further research on the epidemiology of co-infections and their seasonality is needed. The investigation aims to strengthen our understanding of which bacteria and viruses are more likely to occur as part of respiratory co-infections, which co-infection pairings appear more or less frequently than expected under observed pathogen co-circulation dynamics, and which seasonal patterns they exhibit. We focused our research on a 10-year period before the onset of the COVID-19 pandemic to obtain baseline information on the co-occurrence and dynamics of common respiratory pathogens in patient samples.

## Results

### Patient and sample characteristics

The median (IQR) age of patients was 5.0 (1.0; 52.0) years, with two-thirds being children and one-third adults at the time of sampling (Table 1). With a proportion of 59.7% (9,856/16,520), the sample contained more male than female subjects. While almost half of the samples in children were collected from the upper respiratory tract, the majority of samples in adults originated from the lower respiratory tract (LRT). These descriptive results differed minimally between the full analysis set and the analysis set used to evaluate pairwise co-infections and seasonality.

**Table 1:** Patient and sample characteristics with detection results by age group

	Total (*n.*, %)		Children (0–17 years)		Adults (≥18 years)		*p* value^a^
	16,520	100.0%	10,904	66.0%	5,616	34.0%	
Sex							
Male	9,856	59.7%	6,362	58.3%	3,494	62.2%	<0.001
Female	6,664	40.3%	4,542	41.7%	2,122	37.8%	
Postal code							
86XXX	13,747	83.2%	8,649	79.3%	5,098	90.8%	<0.001
85XXX	276	1.7%	236	2.2%	40	0.7%	
Others	2,497	15.1%	2,019	18.5%	478	8.5%	
Sample location							
Pharynx/nose/mouth	6,087	36.8%	5,070	46.5%	1,017	18.1%	<0.001
Bronchi/alveoli/trachea	3,396	20.6%	292	2.7%	3,104	55.3%	
Others	103	0.6%	40	0.4%	63	1.1%	
Not specified	6,934	42.0%	5,502	50.5%	1,432	25.5%	
Infection result							
All negative	9,848	59.6%	6,191	56.8%	3,657	65.1%	<0.001
Mono-infection	5,459	33.0%	4,008	36.8%	1,451	25.8%	
Co-Infection	1,213	7.3%	705	6.5%	508	9.0%	
Co-infection subclasses							
Dual-infection	966	79.6%	533	75.6%	433	85.2%	<0.001
Tri-infection	198	16.3%	132	18.7%	66	13.0%	
Quad^+^-infection	49	4.0%	40	5.7%	9	1.8%	
Co-infection combination type							
Interviral co-infection	388	32.0%	360	51.1%	28	5.5%	<0.001
Interbacterial co-infection	563	46.4%	238	33.8%	325	64.0%	
Viral-bacterial co-infection	262	21.6%	107	15.2%	155	30.5%	

All tests were conducted at the University Hospital of Augsburg. The postal code corresponds to residences in Germany, particularly around the Augsburg region.

Based on full dataset after exclusion of patients with fully non-interpretable test results from December 27, 2007 to May 11, 2018.

a Pearson’s chi-squared test.

The testing regimen varied considerably between age groups and over time. First, the number of annually performed tests tended to increase over the observation period (Figure S1). Second, the diagnostic strategy had evolved from pathogen-specific to broader testing from 2008 to 2011. Third, markedly more viral than bacterial tests were performed per patient in children, whereas the ratio of viral to bacterial tests was relatively balanced in adults (Table S1). Given the different relevance of pathogens and testing strategies across age groups, all further results were stratified accordingly.

### Overall co-infections

Of all screened patients, 7.3% (1,213/16,520) presented a co-infection. Although the test-positive rate for a co-infection was higher among adults (9.0%, 508/5,616) than among children (6.5%, 705/10,904), controlling for the number of pathogen tests per patient reveals higher chance of children presenting with co-infection (*p* < 0.001). The frequency of co-infections among children was interviral, whereas among adults nearly two-thirds involved only bacteria. However, particularly for children, this ratio should be interpreted with caution given their unequal viral-bacterial testing regimen.

Similar to the underlying sample, there were significantly more male patients testing positive for a (co-)infection regardless of age group (*p* < 0.001 each) (Tables S2 and S3). This sex imbalance is greatest in co-infected adults [male: 66.5% (338/508)], with a statistically significant difference (*p* = 0.012) from adults who tested negative or positive for one pathogen. The proportion of co-infections based on samples collected in the LRT was 6.4% (45/705) in children and 72.0% (366/508) in adults, each higher than for the respective mono-infections (*p* < 0.001 each).

### Pathogen-specific co-infections

In both adults and children, *H. influenzae and S. pneumoniae* were the most prevalent bacteria in general (Tables S4–S6). They were also by far the most common bacteria occurring as part of a co-infection, regardless of patient age. Among viral infections, influenza A virus (IAV) was most common in adults and respiratory syncytial virus (RSV) in children, followed by human rhinovirus (hRV) in both age groups. However, hRV was the most prominent virus involved in co-infections among both, children and adults.

Looking at bacterial co-infection rates, *S. pneumoniae* ranked first in children and *M. catarrhalis* in adults (considering only pathogens with at least 20 positive detections over the entire observation period). Among viruses, adenovirus (AdV) presented the highest co-infection rate in both age groups. Conversely, the lowest co-infection rates among bacteria were observed for *B. pertussis* in children and *L. pneumophila* in adults, and among viruses for RSV and influenza B virus (IBV) in children and adults, respectively.

### Pairwise co-infections

**Figure 1:**
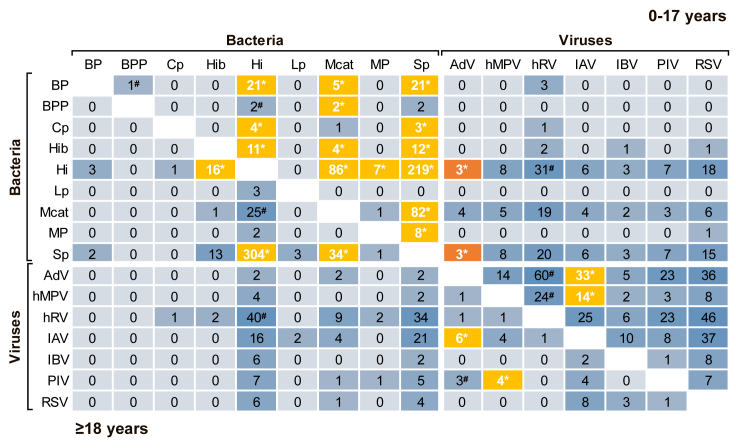
Observed cumulative incidences of pairwise co-infections by age group from 2008 to 2017 The main diagonal of the matrix separates incidences into pairwise co-infections among children (upper right triangle) and adults (lower left triangle). The blue color gradient represents level of observed cumulative incidences. Statistically significant (∗*p* ≤ 0.05), higher observed than expected cumulative incidences according to the proportional distribution model are marked yellow, whereas lower observed than expected cumulative incidences are marked orange. For incidences with hashtag, sensitivity analysis revealed no statistical significance for at least one comparison in the model. AdV, adenovirus; BP, *B. pertussis*; BPP, *B. parapertussis*; Cp, *Chl. pneumoniae*; Hi, *H. influenzae*; Hib, *H. influenzae type b*; hMPV, human metapneumovirus; hRV, human rhinovirus; IAV, influenza A virus; IBV, influenza B virus; Lp, *L. pneumophila*; Mcat, *M. catarrhalis*; MP, *M. pneumoniae*; PIV, parainfluenza virus 1, 2, 3; RSV, respiratory syncytial virus; Sp, *S. pneumoniae*.

Among bacteria, *H. influenzae*, *S. pneumoniae*, *and M. catarrhalis* had a considerable tendency to co-infect with other pathogens (Figure 1). The proportional distribution model likewise revealed higher than expected incidences for many of their co-infection pairings, especially between each other. Although hRV frequently occurred as part of a co-infection, the model revealed that only its pairwise occurrence with AdV and human metapneumovirus (hMPV) in children and *H. influenzae* in both age groups deviated from expected co-infection incidences, all of which did not reach statistical significance in sensitivity analysis. Generally, interviral co-infections were common among children, especially with RSV and AdV besides hRV. However, after sensitivity analysis, only pairings of IAV with AdV and IAV with hMPV remained significant. In contrast, AdV in children presented a lower observed than expected pairwise incidence with *H. influenzae* and *S. pneumoniae*.

**Figure 2:**
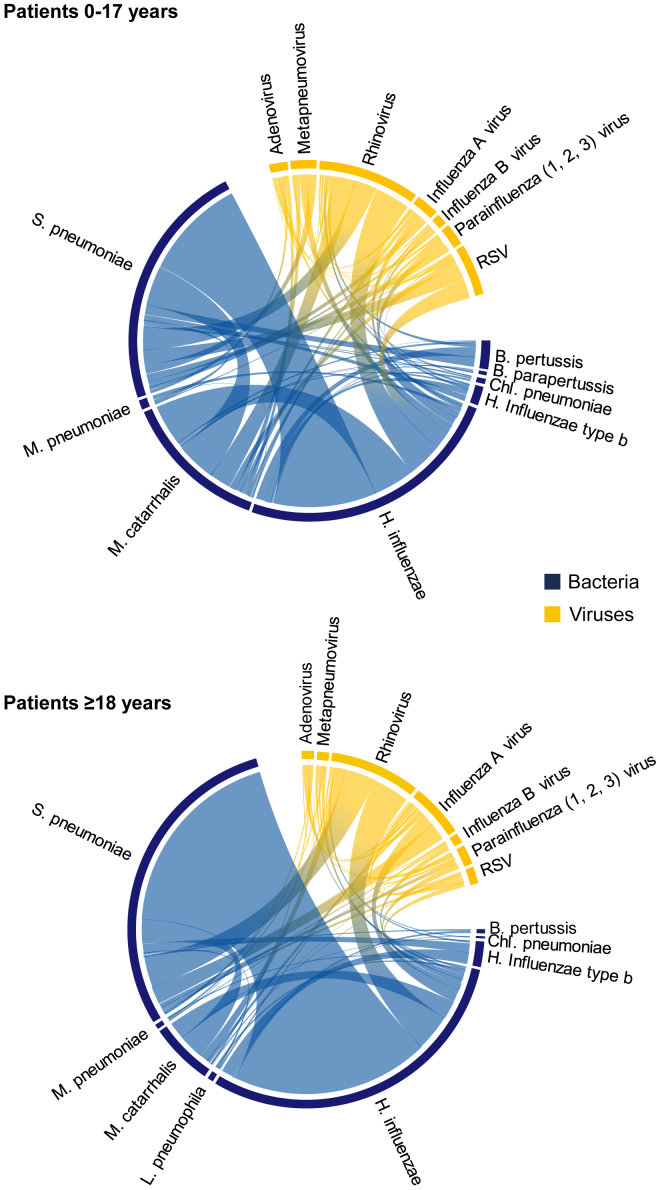
Chord diagram on the frequency of pathogens to occur as part of co-infections in children up to 17 years and in adults at the Augsburg University Hospital from December 27, 2007 to May 11, 2018

Figure 2 visualizes the results on pathogen-specific co-infection frequencies and the most common pairwise co-infections in adults (Figure 2A) and children (Figure 2B). Therein, the proportion of a pathogen at the perimeter of the circle represents the share of that pathogen among all pairwise co-infections. The proportion of each co-infection band in the circumferential fraction of a pathogen further represents the share of that co-infection pairing in all pairwise co-infections of the respective pathogen.

**Figure 3:**
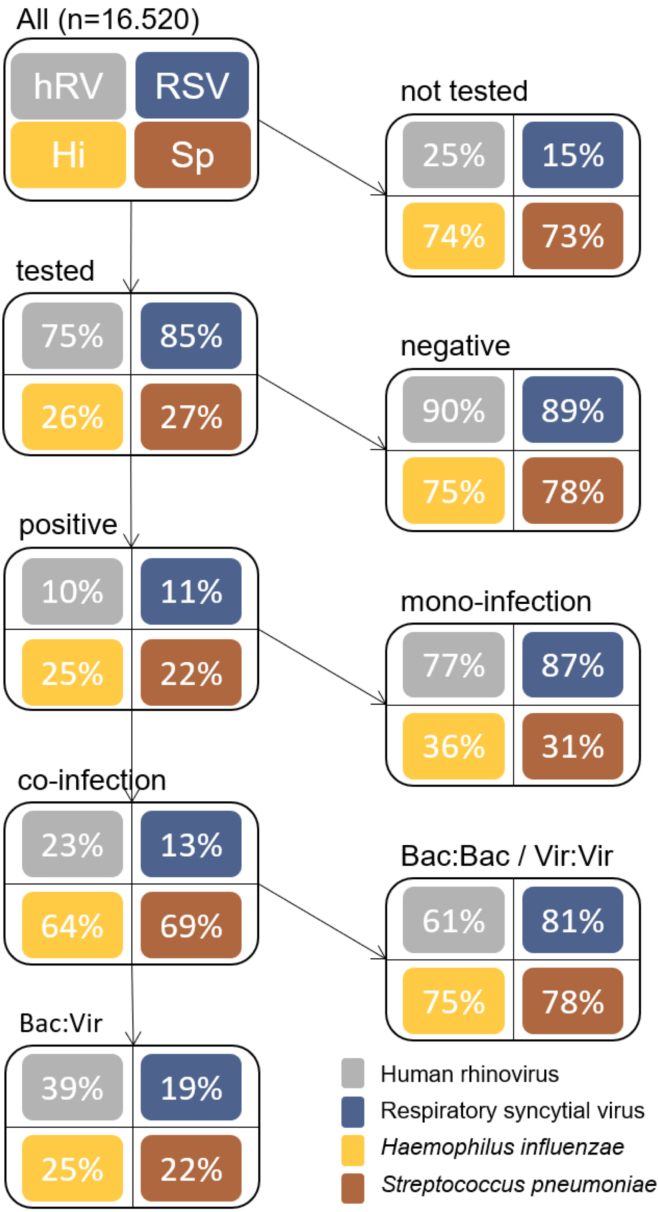
Diagnostic results for all pathogens for which >10% of samples tested positive among patients of all ages at the Augsburg University Hospital from December 27, 2007 to May 11, 2018 hRV, human rhinovirus; RSV, respiratory syncytial virus; Hi, *H. influenzae*; Sp, *S. pneumoniae*.

Without regard to age, an overview of co-infection frequencies for all pathogens for which more than 10% of samples tested positive is provided in Figure 3 to highlight co-infections with high incidences.

### Seasonality of co-infections

**Figure 4:**
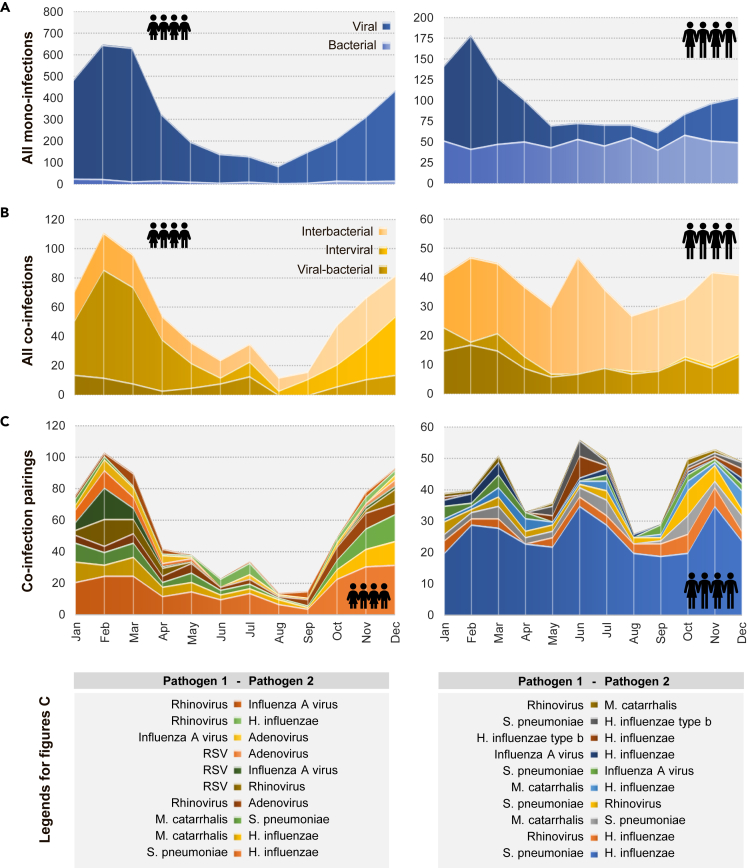
Monthly cumulative incidences of all mono-infections (A) All co-infections (B) and ten most common co-infection pairings (C) by age group, shown by pictograms for children (0-17 years) and adults (≥ 18 years) from 2008 to 2017.

On average, co-infection frequencies showed a similar seasonality across age groups when compared to mono-infections (Figure S2). Specifically, both mono- and co-infections peaked in winter and were less common in summer. Furthermore, the intensity of seasonality in mono- and co-infections decreased with age and was lower for co-infections than for mono-infections. The reduced seasonality of co-infections in adults mainly resided in a substantial and annually recurrent peak in co-infections during the summer months (around June), which was primarily driven by interbacterial co-infections, particularly between *H. influenzae* and *S. pneumoniae* (Figure 4). The samples underlying these co-infections originated primarily from the LRT (Figure S3). In comparison, the average annual course of co-infections in children followed a different pattern. The much stronger seasonality in children was largely driven by interviral co-infections with RSV involvement during the winter and less detections of bacterial co-infections during the summer. However, a summer peak in cumulative co-infections was also observed, which was less pronounced than in adults and was attributable to co-infections of *H. influenzae* with *S. pneumoniae* or hRV. Examination of cumulative test-positive rates revealed a more pronounced peak also in children during spring/summer (Figure S4).

## Discussion

A recent study on the co-circulation of influenza virus and severe acute respiratory syndrome coronavirus 2 (SARS-CoV-2) illustrated the potentially serious public health threat of co-infections.^12^ In our study, we analyzed a large, 10-year dataset to obtain information on the circulation dynamics and frequencies of respiratory pathogens under baseline conditions. Certain co-infection pairings were found to occur at unexpected frequency and, interestingly, co-infections exhibited a pronounced summer peak, especially between *H. influenzae* and *S. pneumoniae* among adults.

The observed overall proportion of co-infections was similar to that reported by other studies with comparable populations.^4^^,^^13^ After adjusting for the number of pathogen tests per patient, children were more likely to be co-infected than adults, which had been observed previously and discussed to be associated with their more immature immune systems, greater social interaction, and different pathogen diversity.^4^^,^^10^ Moreover, the sex imbalance in respiratory co-infections observed here is consistent with past findings, although the present proportion of males among co-infected adults of nearly two-thirds exceeds that of previous observations.^3^^,^^14^ Greater susceptibility of men to respiratory infections is linked to immunological, anatomic, lifestyle, behavioral, and socioeconomic factors.^15^ Although no clinical data were available for our analysis, the higher numbers of co-infections detected in the LRT compared to mono-infections in both age groups may be indicative of increased disease severity in co-infected patients.

The frequent co-occurrence of *H. influenzae* and *S. pneumoniae* was not unexpected, as their bidirectional interference with both bacteria and viruses has been extensively documented.^7^^,^^16^^,^^17^ *S. pneumoniae* and *H. influenzae* are actually well-adopted commensals of the human nasopharynx,^18^ which raises the question whether the frequent co-occurrence in our samples might be an epiphenomenon. The nasal mucosal immune system normally controls the spread of these bacteria,^19^ but when normal immunosurveillance mechanisms are disturbed, they can migrate and colonize different niches of the upper and lower respiratory tract where they are frequently found as part of bacterial biofilms under pathological conditions.^20^ Despite the introduction of vaccination against pneumococci, co-infections with *S. pneumoniae* but also *H. influenzae* continue to pose a significant health risk, being frequently involved in secondary bacterial otitis media, sinusitis, and pneumonia.^21^^,^^22^ Co-infection dynamics can be changed due to novel pathogens such as SARS-CoV-2. Recently, Pipek et al.^23^ published that in total 7,700 co-infection samples were detected out of 2 million samples from the database.

The present study further demonstrated a high prevalence of hRV among co-infections, likely owing to the reduced local immunocompetence caused by hRV.^24^ hRV co-infections have in turn been proposed to worsen respiratory illnesses, including exacerbations of asthma.^25^ However, AdV presented the highest co-infection rates among viruses in both age groups, contrasting with RSV and IBV which reported the lowest in children and adults, respectively. While the outcomes on AdV and IBV are consistent with past findings,^4^^,^^6^^,^^26^ RSV tended to be in the middle/upper range of co-infection rates in other publications.^4^^,^^27^ Surprisingly, and in contrast to preceding evidence,^5^^,^^6^^,^^26^ IAV was detected relatively often with other viruses, especially in children. One potential reason for this could be IAV vaccination rates in Germany, which are still considered too low—most notably in Bavaria.^28^ The resulting greater IAV community circulation puts vulnerable groups, such as children, at higher risk of getting diseased.^29^

Some pathogen pairings revealed unexpected incidences when compared to model-based expectations. These results indicate that, in accordance with the current state of research,^4^^,^^6^ pathogens do not simply co-occur at random, but that synergistic and competitive mechanisms as outlined previously are involved in co-infection dynamics.^1^^,^^16^ Notably, clustered and unexpectedly high co-occurrences between *H. influenzae*, *S. pneumoniae*, and *M. catarrhalis* were seen in both age groups. High polymicrobial colonization rates of these species have been linked to their partially synergistic interplay, in particular their formation of multispecies biofilms potentially enhancing bacterial persistence and antibiotic protection.^16^^,^^30^ Among interviral pairings, an unexpectedly high incidence of hRV with AdV was evident in children, while almost completely absent in adults. This finding is in agreement with prior reports^4^^,^^26^^,^^31^^,^^32^ and has been associated with increased disease severity.^31^ However, the underlying interference mechanism and full clinical implication of this pairing remain to be clarified. Interestingly, AdV in children presented a lower pairwise incidence with *H. influenzae* and *S. pneumoniae* than expected. *In vitro* studies have reported reduced cytokine production as a result of AdV infection after initial bacterial exposure^17^ and decreased AdV transduction after inoculation of epithelial cells with *S. pneumoniae*.^33^ While inhibitory effects between AdV and *S. pneumoniae* were suggested based on an epidemiologic investigation, such mechanisms were less consistent *in vivo* for AdV co-occurring with *H. influenzae*.^32^^,^^34^

The higher incidence of co-infections during the winter was in line with the typical seasonal spread of multiple respiratory pathogens, which is driven by changes in the environment, human behavior, and pathogen factors.^35^ The lower seasonality of co-infections compared to mono-infections was presumably owing to competitive pathogen interferences such as pan-antiviral host immunization,^4^ which may attenuate the occurrence of pronounced co-infection peaks. In addition, the differences in seasonality across age groups could be explained by age-specific pathogen circulation patterns, testing practices, or under-reporting. Surprisingly, a substantial summer peak in interbacterial co-infections among adults occurred, predominantly between *H. influenzae* and *S. pneumoniae*. Since neither of the bacteria independently exhibited any distinct summer peak, factors such as favorable synergistic (environmental) conditions, the absence of other competing pathogens, or greater occurrence of other *S. pneumoniae* strains during the summer may explain this observation.

Despite the need for further large-scale controlled trials on molecular mechanisms, epidemiology, and clinical relevance of strain-specific respiratory co-infections, the present findings contribute to a growing body of evidence on a complex phenomenon of great public health concern. Future prospective trials will help to understand dynamic of the co-infections. Moreover, new data also including SARS-CoV-2 detections will give important insights into changes in co-infection dynamics in the presence of a novel pathogen. This study shows that some co-infection patterns are similar across different regions of the respiratory tract, while others are not. Hence, our findings may help adapting future testing strategies, e.g., most of the seasonality of the mono- and co-infections are known; it can reduce the over testing, if the co-infections are not expected; research, e.g., the first pathogen of the co-infection might be also important for the immune response; or policy guidance, e.g., as pandemic preparedness but also countries like India worked on guidelines on prevention and treatment of co-infections of COVID-19 with other diseases like Seasonal Influenza (H1N1). With growing globalization, shrinking space between wildlife and humans, the spread of antibiotic resistances, changing climatic conditions, and the threat of emerging pandemics, continued research on respiratory co-infections appears steadily important.

### Limitations of the study

This study has several limitations. Firstly, since this is a retrospective, monocentric study, conclusions about causality are not possible and representativeness for other regional contexts may not be given. For instance, environmental variables, herd immunity/vaccination rates, or population characteristics can differ significantly, all of which may affect co-infection incidences. Second, it was not possible to discriminate between patients who entered the dataset more than once, although this appeared to have little impact given the large sample size. Third, the applied pathogen detection kits were incapable of distinguishing between many pathogen sub-strains and did not cover all relevant respiratory pathogens (e.g., *S. aureus* or coronavirus). Nonetheless, the number of observed pathogens was comparatively high and comprised bacteria and viruses, this being major strength of the present investigation. Furthermore, the long observation period of more than 10 years and the high number of assay results imply good confidence in the presented findings.

## STAR★Methods

### Key resources table

**Table:** 

REAGENT or RESOURCE	SOURCE	IDENTIFIER
**Deposited data**		

Analyzed data	Laboratory pathogen data from outpatients and inpatients	Augsburg University Hospital between December 27, 2007, and May 11, 2018

**Software and algorithms**		

R Software version 2021.09.2	R Core Team	https://www.r-project.org/
Microsoft® Excel (16.54)	Microsoft®	https://www.microsoft.com/de-de

### Resource availability

#### Lead contact

Further information and requests for resources and reagents should be directed to and will be fulfilled by the lead contact, Mehmet Gökkaya (mehmet.goekkaya@med.uni-augsburg.de).

#### Materials availability

This study did not generate new unique reagents.

#### Data and code availability

All deposited data reported in this paper will be shared by the lead contact upon request. Any additional information required to reanalyze the data reported in this paper is available from the lead contact upon request. All original code has been deposited in the section of STAR methods: quantification and statistical analysis and is publicly available as of the date of publication.

### Experimental model and study participant details

This exploratory study uses pathogen data from outpatients and inpatients tested at the Augsburg University Hospital between December 27, 2007, and May 11, 2018, for a suspected respiratory tract infection. Along with each test result, information was available on the time of sample collection, sample type (e.g., pharyngeal/nasal swab, bronchoalveolar lavage), sex, age, and postal code area of the patient undergoing the test.

Of the original 16,531 test results, 11 were excluded from subsequent analyses due to fully non-interpretable assay results. Test results with partially interpretable (‘weakly positive’, ‘questionably positive’ and ‘questionable’) outcomes were included. In total, 16,520 pathogen detection results were categorized as ‘positive’ for further analyses. Any test result with at least two positive viral or bacterial detections was considered a co-infection. Because different pathogens vary in epidemiological and clinical relevance depending on age, this variable was subdivided for further analyses into the categories of children (0–17 years) and adults (≥18 years). To facilitate the approximate classification of the infection localization regarding lower respiratory tract (LRT) or upper respiratory tract (URT), expressions of the open variable 'sample type' were re-classified into four groups of sampling location.

For identification of pathogens, the reverse hybridization kits AID CAP Bac, AID CAP Vir, and AID Bordetella pertussis with upstream PCR (AID Autoimmun Diagnostika GmbH, Strassberg), as well as an R-DiaRhino PCR test for rhinovirus (Mikrogen GmbH, Neuried), were applied. A total of 16 pathogens were detected with these CE labeled test systems: *B. pertussis, B. parapertussis, Chl. pneumoniae, H. influenzae, H. influenzae type b, L. pneumophila, M. catarrhalis, M. pneumoniae,* and *S. pneumoniae* among bacteria as well as parainfluenza virus 1, 2, 3 (PIV), respiratory syncytial virus (RSV), human metapneumovirus (hMPV), human rhinovirus (hRV), influenza A virus (IAV), influenza B virus (IBV) and adenovirus (AdV) among viruses. Ethical approval was waived as a retrospective analysis of anonymized patient data (Beratungskommission für klinische Forschung; 2018-12; 14th May 2018).

### Method details

The proportional distribution model of Mandelia et al.^4^ (2021) was adopted to determine whether pairwise co-infections occurred more or less frequently than if there was no predisposition to increased or decreased pairwise occurrence. Its central idea lies in the assumption that a pathogen is equally likely to co-infect with any other pathogen circulating simultaneously in the community during a given period of time (i.e., there is no predisposition to increased or decreased pairwise occurrence). For this purpose, two expected incidences are calculated for each co-infection pairing per month based on observed pathogen circulations during that month as well as the overall co-infection rates of the two pathogens involved in the considered pairing. The obtained monthly expected incidences are then accumulated over the full observation period and each compared to the observed cumulative incidence. In our study analysis, two expected incidences were calculated for each pathogen pairing based on constant pathogen co-infection rates in combination with monthly community co-circulation patterns. Formula for the calculation of expected number of co-infections pairing^4^:

${f\left(x,y\right)}_{m}$ Expected number of coinfections of virus/bacteria x by virus/bacteria y,

${CR}_{x}$ Coinfection rate of virus/bacteria x,

$O_{x}$ Count of observed occurrences of virus/bacteria x,

$O_{y}$ Count of observed occurrences of virus/bacteria y,

$O_{total}$ Count of observed occurrences of all virus and bacteria,

m Month,

N Total number of months,

$${f\left(x,y\right)}_{m}=\left\{\left[{CR}_{x}\times {\left(O_{x}\right)}_{m}\right]\times {\left[\frac{O_{y}}{O_{total}-\;O_{x}}\right]}_{m}\right\}$$

$$E{\left(x,y\right)}_{total}=\sum f{\left(x,y\right)}_{m1}+f{\left(x,y\right)}_{m2}+\ldots +f{\left(x,y\right)}_{mN}$$

To examine pairwise co-infection frequencies and their seasonal patterns, cumulative incidences were calculated over the years 2008 through 2017, thereby including only the years for which pathogen detection data were available over the entire course of the respective year. Consequently, the sample size for these analyses was reduced from 16,520 to 15,168 test results. This was to minimize an unequal probability of highly seasonal pathogens entering the analysis set. To account for the potential impact of testing regimens, pairwise co-infection frequencies were additionally analyzed using test-positive rates.

### Quantification and statistical analysis

For comparisons between age groups and infection types Pearson’s Chi-squared test, Fisher’s exact test and two-proportions z-test were used. Expected estimates were then respectively compared to the observed pairwise incidence using exact binomial tests. Only when statistical significance was found for both comparisons, a co-infection pairing was considered of interest. As a sensitivity analysis, the model was re-calculated using the lower and upper 95% confidence interval limits of co-infection rates. For all analyses, Microsoft Excel (16.54) or R (2021.09.2) were used. Excel file can be provided upon request (Data S1).

## Supplemental information

Document S1. Figures S1–S4 and Tables S1–S6

Data S1. Excerpt of the R script for the data analysis
