# Supplementary material for: Ten-year retrospective data analysis reveals frequent respiratory co-infections in hospitalized patients in Augsburg
Source: iScience. 2024 May 28;27(6):110136. doi: 10.1016/j.isci.2024.110136 (PMC11223076; doi:10.1016/j.isci.2024.110136)
Supplement: Document S1. Figures S1–S4 and Tables S1–S6 [file mmc1.pdf]

iScience, Volume *27*

Supplemental information

Ten-year retrospective data analysis reveals frequent respiratory co-infections in hospitalized patients in Augsburg

Martin Krammer, Reinhard Hoffmann, Hans-Georg Ruf, Avidan U. Neumann, Claudia Traidl-Hoffmann, Mehmet Goekkaya, and Stefanie Gilles

Supplementary material

Figure S1: Annual tests number, related to the results about patient and sample characteristics. Annual number of conducted respiratory bacterial (A) and viral (B) tests at the Augsburg University Hospital.

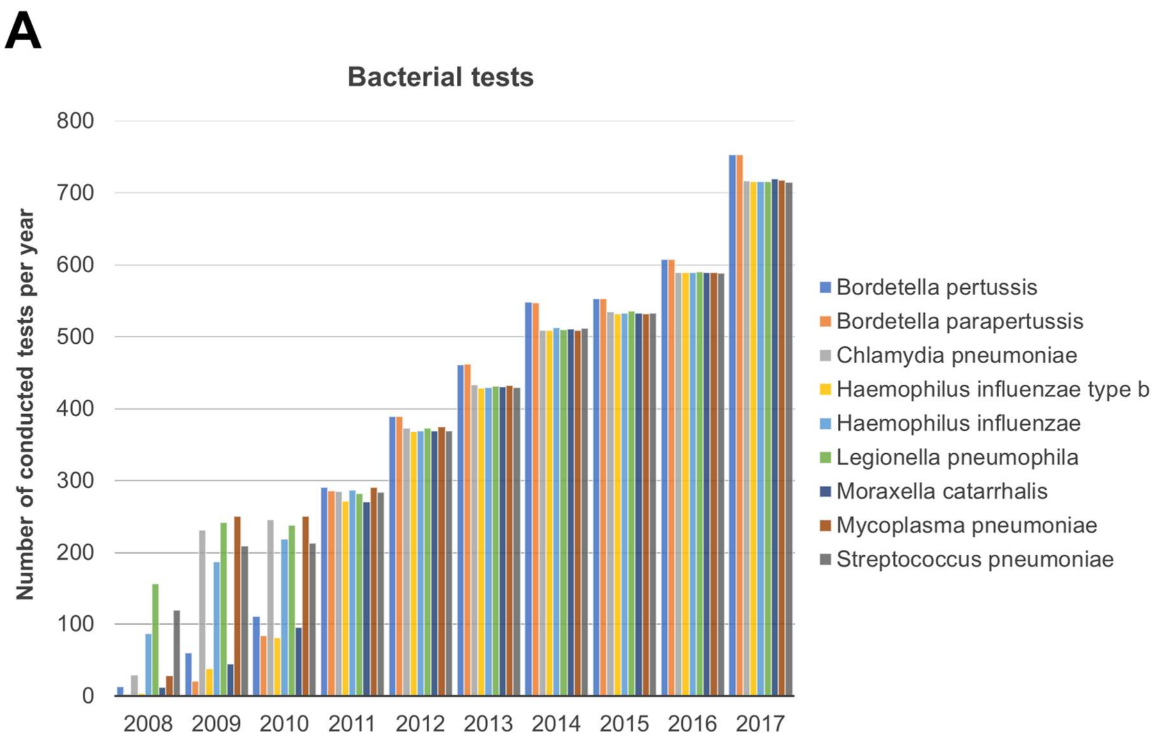

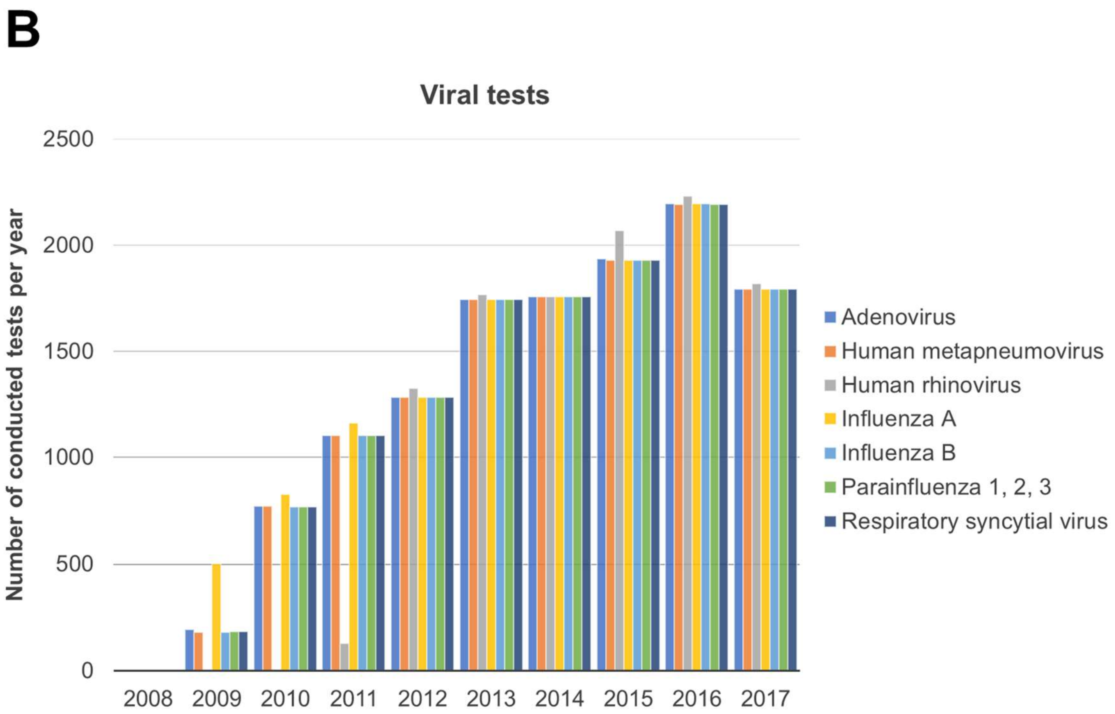

Figure S2: Seasonality of mono- and co-infections by age group with summer as reference season, related to results about seasonality of co-infections. Cumulative incidences from 2008 to 2017; Meteorological seasons for Augsburg, Bavaria: winter (01/12 – 27/02), spring (01/03 – 31/05), summer (01/06 – 31/08) and autumn (01/09 – 30/11).

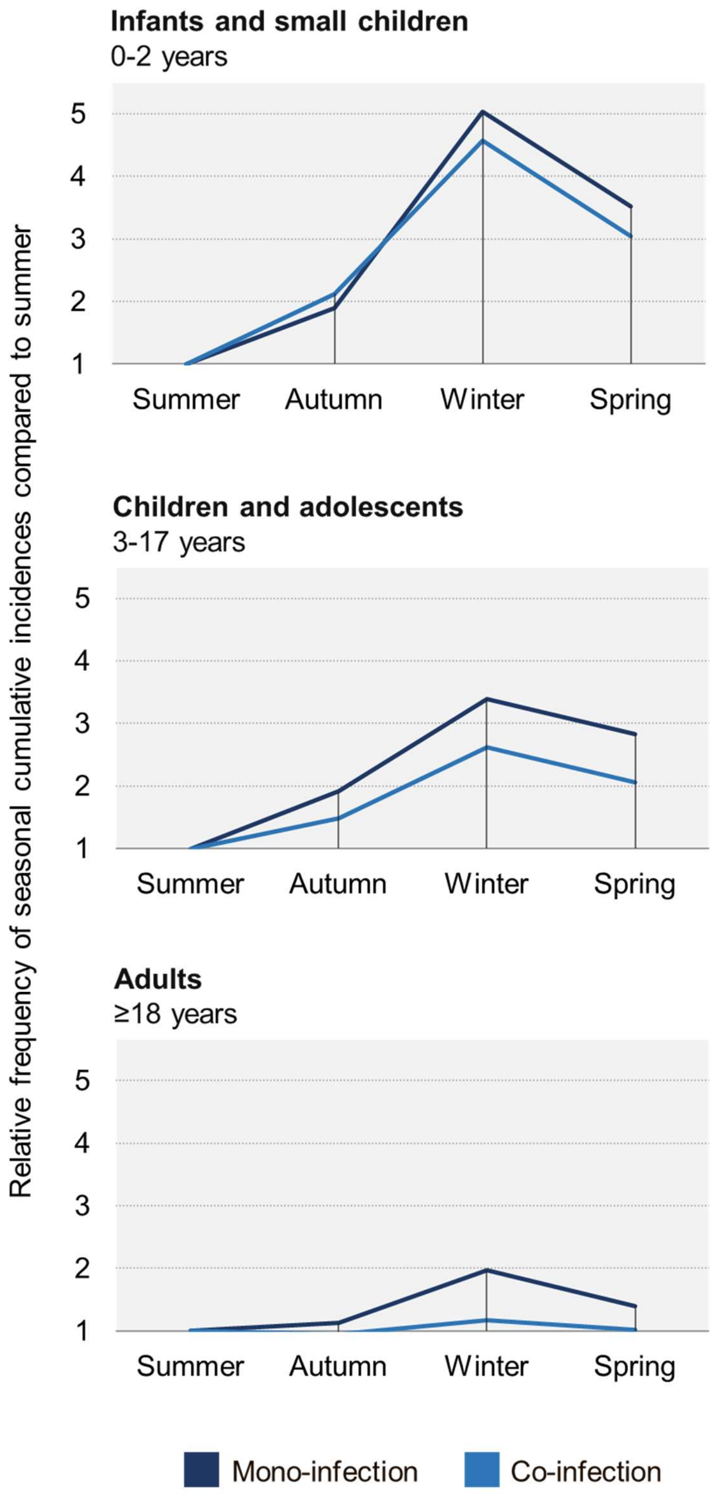

Figure S3: Monthly absolute (co-)infection incidences with sample location by age group from 2013 to 2017, related to results about seasonality of co-infections.

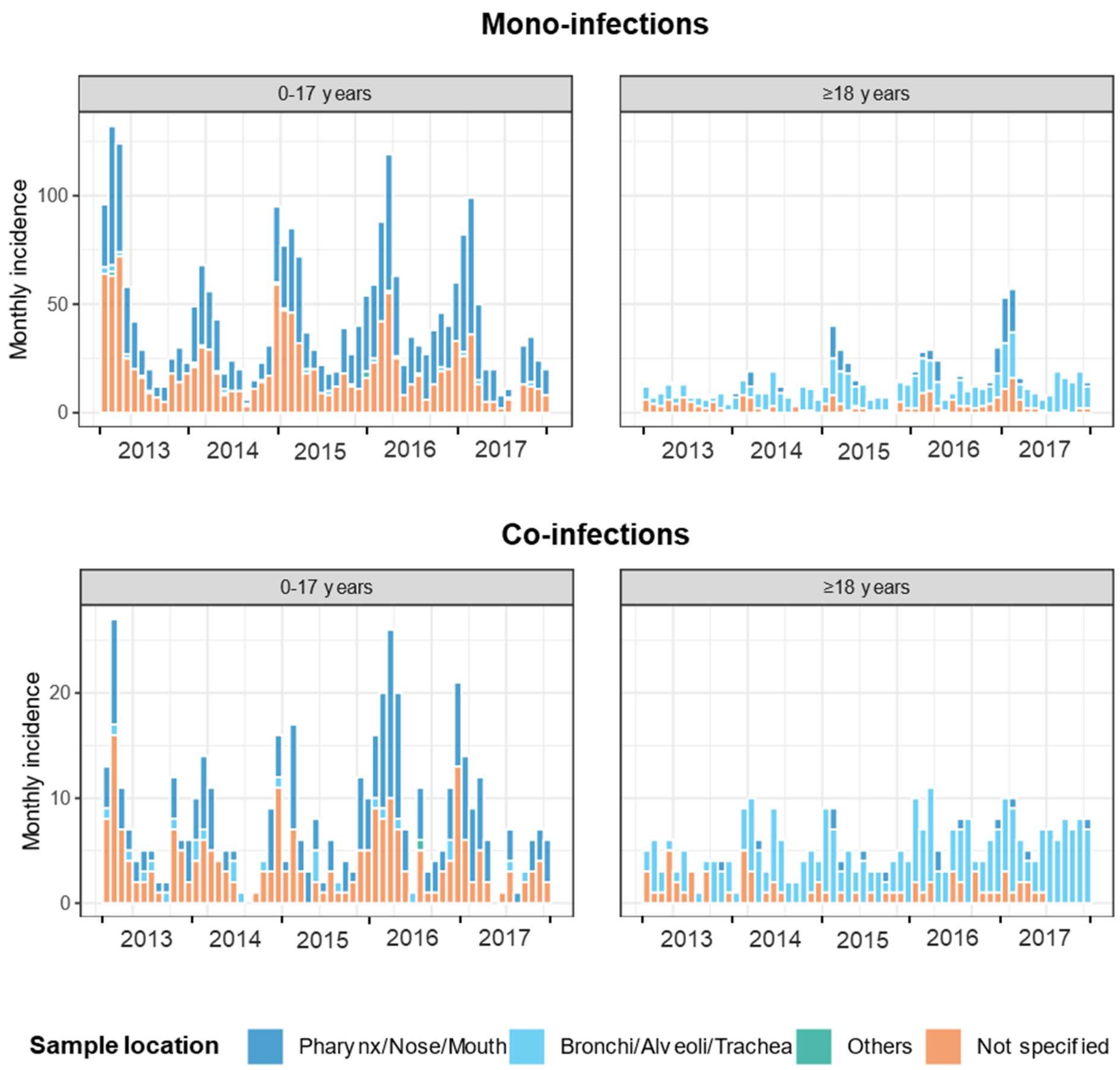

Figure S4: Monthly cumulative test-positive rates of ten most common pairwise co-infections from 2008 to 2017, related to results about seasonality of co-infections.

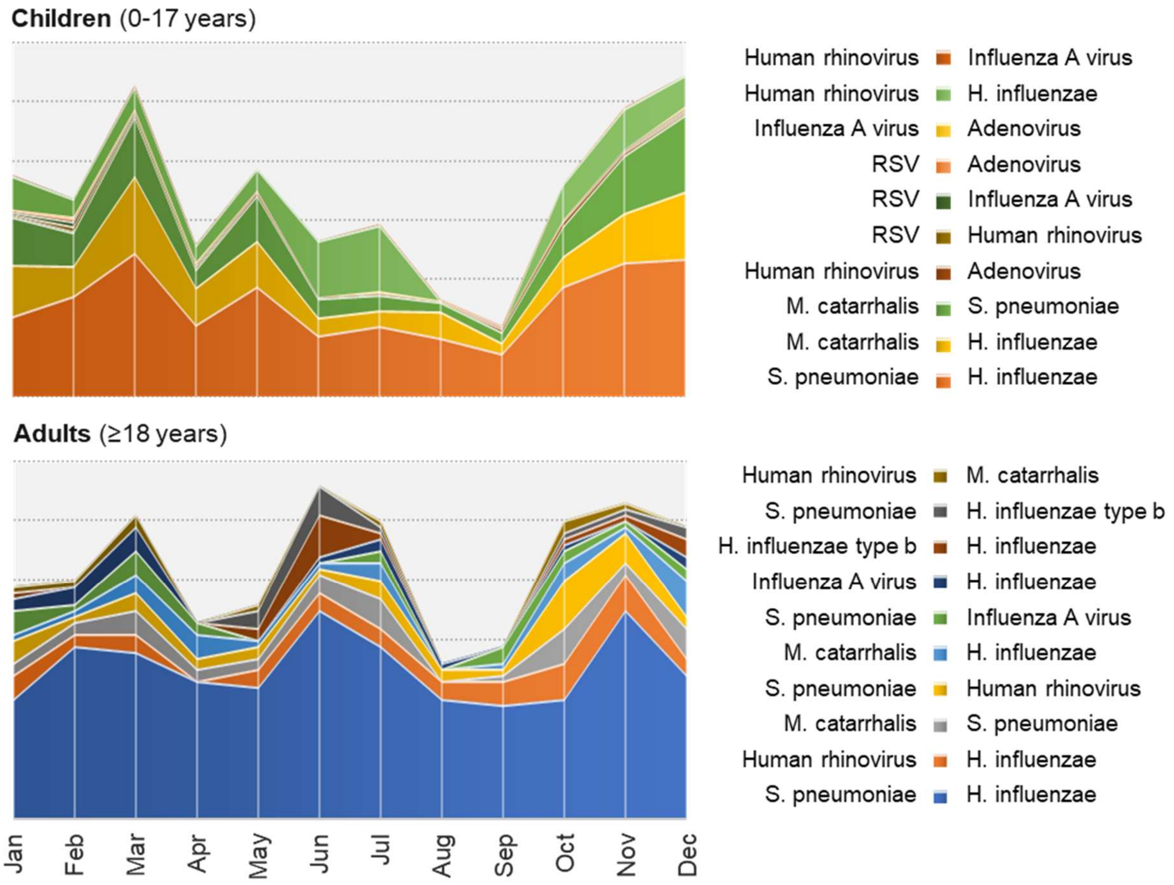

Table S1: Average number of pathogens tested for per patient, related to the results about patient and sample characteristics.

	Total	Patients 0-17 years	Patients ≥18 years
Tests for viral pathogen	5.88	6.10	5.43
Tests for bacterial pathogen	2.31	0.62	5.60
Total	8.19	6.72	11.04

Table S2: Comparison of sex and sampling location between infection types for patients 0-17 years, related to the results about overall co-infections.

	All negative		Mono-infection		Co-infection		p-value
	6,191	56.8%	4,008	36.8%	705	6.5%	
Male	3,573	57.7%	2,384	59.5%	405	57.4%	0.20*
Female	2,618	42.3%	1,624	40.5%	300	42.6%	
Pharynx/nose/mouth	2,955	47.7%	1,810	45.2%	305	43.3%	<0.001**
Bronchi/alveoli/trachea	181	2.9%	66	1.6%	45	6.4%	
Others	31	0.5%	8	0.2%	1	0.1%	
Not specified	3,024	48.8%	2,124	53.0%	354	50.2%	

* Pearson's Chi-squared test
** Fisher's exact test with simulated p-value (based on 2000 replicates)

Table S3: Comparison of sex and sampling location between infection types for patients ≥18 years, related to the results about overall co-infections.

	All negative		Mono-infection		Co-infection		p-value*
	3,657	65.1%	1,451	25.8%	508	9.0%	
Male	2,282	62.4%	874	60.2%	338	66.5%	0.039
Female	1,375	37.6%	577	39.8%	170	33.5%	
Pharynx/nose/mouth	758	20.7%	236	16.3%	23	4.5%	<0.001
Bronchi/alveoli/trachea	1,886	51.6%	852	58.7%	366	72.0%	
Others	60	1.6%	3	0.2%	0	0.0%	
Not specified	953	26.1%	360	24.8%	119	23.4%	

* Pearson's Chi-squared test

Table S4: Diagnostic results for respiratory tract infections among patients of all ages at the Augsburg University Hospital, related to results pathogen-specific co-infections.

	BACTERIA									VIRUSES						
	Bordetella pertussis	*Bordetella parapertussis*	*Chlamydia pneumoniae*	*Haemophilus influenzae type b*	*Haemophilus influenzae*	*Legionella pneumophila*	*Moraxella catarrhalis*	*Mycoplasma pneumoniae*	*Streptococcus pneumoniae*	*Adenovirus*	*Human meta-pneumovirus*	*Human rhinovirus*	*Influenza A virus*	*Influenza B virus*	*Parainfluenza virus 1, 2, 3*	*Respiratory syncytial virus*
Not tested	12,318	12,396	12,160	12,572	12,177	12,027	12,534	12,131	12,132	2,459	2,481	4,127	2,051	2,484	2,484	2,479
	74.6%	75.0%	73.6%	76.1%	73.7%	72.8%	75.9%	73.4%	73.4%	14.9%	15.0%	25.0%	12.4%	15.0%	15.0%	15.0%
Tested	4,202	4,124	4,360	3,948	4,343	4,493	3,986	4,389	4,388	14,061	14,039	12,393	14,469	14,036	14,036	14,041
	25.4%	25.0%	26.4%	23.9%	26.3%	27.2%	24.1%	26.6%	26.6%	85.1%	85.0%	75.0%	87.6%	85.0%	85.0%	85.0%
Negative	4,138	4,112	4,341	3,887	3,255	4,437	3,732	4,347	3,406	13,391	13,578	11,128	13,506	13,639	13,568	12,531
	98.5%	99.7%	99.6%	98.5%	74.9%	98.8%	93.6%	99.0%	77.6%	95.2%	96.7%	89.8%	93.3%	97.2%	96.7%	89.2%
Unusable/wrong material	3	7	9	9	9	11	9	11	11	9	9	1	6	8	8	9
	0.1%	0.2%	0.2%	0.2%	0.2%	0.2%	0.2%	0.3%	0.3%	0.1%	0.1%	0.0%	0.0%	0.1%	0.1%	0.1%
(Weakly, questionably) positive or questionable	61	5	10	52	**1,079**	45	245	31	**971**	661	452	1,264	957	389	460	1,501
	1.5%	0.1%	0.2%	1.3%	**24.8%**	1.0%	6.1%	0.7%	**22.1%**	4.7%	3.2%	10.2%	6.6%	2.8%	3.3%	10.7%
Occurring as mono-infection	28	2	3	18	392	39	55	16	304	488	359	977	771	324	378	1,305
	45.9%	40.0%	30.0%	34.6%	36.3%	86.7%	22.4%	51.6%	31.3%	73.8%	79.4%	77.3%	80.6%	83.3%	82.2%	86.9%
Occurring as part of co-infection (co-infection rate)	33	3	7	34	687	6	190	15	667	173	93	287	186	65	82	196
	54.1%	60.0%	70.0%	65.4%	63.7%	13.3%	77.6%	48.4%	68.7%	26.2%	20.6%	22.7%	19.4%	16.7%	17.8%	13.1%
Occurring as part of bacterial + viral co-infection	3	0	2	6	169	2	66	4	149	12	24	111	52	22	19	38
	9.1%	0.0%	28.6%	17.6%	24.6%	33.3%	34.7%	26.7%	22.3%	6.9%	25.8%	38.7%	28.0%	33.8%	23.2%	19.4%
Occurring as part of either bacterial or viral co-infection	30	3	5	28	518	4	124	11	518	161	69	176	134	43	63	158
	90.9%	100.0%	71.4%	82.4%	75.4%	66.7%	65.3%	73.3%	77.7%	93.1%	74.2%	61.3%	72.0%	66.2%	76.8%	80.6%

Table S5: Diagnostic results for respiratory tract infections among patients aged 0-17 years at the Augsburg University Hospital, related to results pathogen-specific co-infections.

	BACTERIA									VIRUSES						
	Bordetella pertussis	*Bordetella parapertussis*	*Chlamydia pneumoniae*	*Haemophilus influenzae type b*	*Haemophilus influenzae*	*Legionella pneumophila*	*Moraxella catarrhalis*	*Mycoplasma pneumoniae*	*Streptococcus pneumoniae*	*Adenovirus*	*Human meta-pneumovirus*	*Human rhinovirus*	*Influenza A virus*	*Influenza B virus*	*Parainfluenza virus 1, 2, 3*	*Respiratory syncytial virus*
Not tested	9,981	10,036	10,178	10,230	10,180	10,227	10,202	10,167	10,173	1,236	1,246	2,530	1,018	1,249	1,249	1,247
	91.5%	92.0%	93.3%	93.8%	93.4%	93.8%	93.6%	93.2%	93.3%	11.3%	11.4%	23.2%	9.3%	11.5%	11.5%	11.4%
Tested	923	868	726	674	724	677	702	737	731	9,668	9,658	8,374	9,886	9,655	9,655	9,657
	8.5%	8.0%	6.7%	6.2%	6.6%	6.2%	6.4%	6.8%	6.7%	88.7%	88.6%	76.8%	90.7%	88.5%	88.5%	88.6%
Negative	868	861	719	658	353	674	543	722	389	9,027	9,288	7,327	9,274	9,408	9,274	8,242
	94.0%	99.2%	99.0%	97.6%	48.8%	99.6%	77.4%	98.0%	53.2%	93.4%	96.2%	87.5%	93.8%	97.4%	96.1%	85.3%
Unusable/wrong material	1	2	2	2	2	2	2	3	2	7	7	0	5	6	6	7
	0.1%	0.2%	0.3%	0.3%	0.3%	0.3%	0.3%	0.4%	0.3%	0.1%	0.1%	0.0%	0.1%	0.1%	0.1%	0.1%
(Weakly, questionably) positive or questionable	54	5	5	14	**369**	1	157	12	**340**	634	363	1,047	607	241	375	1,408
	5.9%	0.6%	0.7%	2.1%	**51.0%**	0.1%	22.4%	1.6%	**46.5%**	6.6%	3.8%	12.5%	6.1%	2.5%	3.9%	14.6%
Occurring as mono-infection	25	2	0	0	77	1	28	2	60	474	287	829	482	199	309	1,233
	46.3%	40.0%	0.0%	0.0%	20.9%	100.0%	17.8%	16.7%	17.6%	74.8%	79.1%	79.2%	79.4%	82.6%	82.4%	87.6%
Occurring as part of co-infection (co-infection rate)	29	3	5	14	**292**	0	**129**	10	**280**	160	76	**218**	125	**42**	66	175
	**53.7%**	60.0%	100.0%	100.0%	**79.1%**	0.0%	**82.2%**	83.3%	**82.4%**	**25.2%**	20.9%	20.8%	20.6%	17.4%	17.6%	**12.4%**
Occurring as part of bacterial + viral co-infection	3	0	1	4	80	0	46	1	67	6	15	44	11	5	10	28
	10.3%	0.0%	20.0%	28.6%	27.4%	n/a	35.7%	10.0%	23.9%	3.8%	19.7%	20.2%	8.8%	11.9%	15.2%	16.0%
Occurring as part of either bacterial or viral co-infection	26	3	4	10	212	0	83	9	213	154	61	174	114	37	56	147
	89.7%	100.0%	80.0%	71.4%	72.6%	n/a	64.3%	90.0%	76.1%	96.3%	80.3%	79.8%	91.2%	88.1%	84.8%	84.0%

Table S6: Diagnostic results for respiratory tract infections among patients ≥18 years at the Augsburg University Hospital, related to results pathogen-specific co-infections.

	BACTERIA									VIRUSES						
	Bordetella pertussis	*Bordetella parapertussis*	*Chlamydia pneumoniae*	*Haemophilus influenzae type b*	*Haemophilus influenzae*	*Legionella pneumophila*	*Moraxella catarrhalis*	*Mycoplasma pneumoniae*	*Streptococcus pneumoniae*	*Adenovirus*	*Human meta-pneumovirus*	*Human rhinovirus*	*Influenza A virus*	*Influenza B virus*	*Parainfluenza virus 1, 2, 3*	*Respiratory syncytial virus*
Not tested	2,337	2,360	1,982	2,342	1,997	1,800	2,332	1,964	1,959	1,223	1,235	1,597	1,033	1,235	1,235	1,232
	41.6%	42.0%	35.3%	41.7%	35.6%	32.1%	41.5%	35.0%	34.9%	21.8%	22.0%	28.4%	18.4%	22.0%	22.0%	21.9%
Tested	3,279	3,256	3,634	3,274	3,619	3,816	3,284	3,652	3,657	4,393	4,381	4,019	4,583	4,381	4,381	4,384
	58.4%	58.0%	64.7%	58.3%	64.4%	67.9%	58.5%	65.0%	65.1%	78.2%	78.0%	71.6%	81.6%	78.0%	78.0%	78.1%
Negative	3,270	3,251	3,622	3,229	2,902	3,763	3,189	3,625	3,017	4,364	4,290	3,801	4,232	4,231	4,294	4,289
	99.7%	99.8%	99.7%	98.6%	80.2%	98.6%	97.1%	99.3%	82.5%	99.3%	97.9%	94.6%	92.3%	96.6%	98.0%	97.8%
Unusable/wrong material	2	5	7	7	7	9	7	8	9	2	2	1	1	2	2	2
	0.1%	0.2%	0.2%	0.2%	0.2%	0.2%	0.2%	0.2%	0.2%	0.0%	0.0%	0.0%	0.0%	0.0%	0.0%	0.0%
(Weakly, questionably) positive or questionable	7	0	5	38	**710**	44	88	19	**631**	27	89	217	**350**	148	85	93
	0.2%	0.0%	0.1%	1.2%	19.6%	1.2%	2.7%	0.5%	17.3%	0.6%	2.0%	5.4%	7.6%	3.4%	1.9%	2.1%
Occurring as mono-infection	3	0	3	18	315	38	27	14	244	14	72	148	289	125	69	72
	42.9%	n/a	60.0%	47.4%	44.4%	86.4%	30.7%	73.7%	38.7%	51.9%	80.9%	68.2%	82.6%	84.5%	81.2%	77.4%
Occurring as part of co-infection (co-infection rate)	4	0	2	20	**395**	6	61	5	**387**	13	17	**69**	61	23	16	21
	57.1%	n/a	40.0%	52.6%	55.6%	13.6%	**69.3%**	26.3%	61.3%	**48.1%**	19.1%	31.8%	17.4%	15.5%	18.8%	22.6%
Occurring as part of bacterial + viral co-infection	0	0	1	2	89	2	20	3	82	6	9	67	41	17	9	10
	0.0%	n/a	50.0%	10.0%	22.5%	33.3%	32.8%	60.0%	21.2%	46.2%	52.9%	97.1%	67.2%	73.9%	56.3%	47.6%
Occurring as part of either bacterial or viral co-infection	4	0	1	18	306	4	41	2	305	7	8	2	20	6	7	11
	100.0%	n/a	50.0%	90.0%	77.5%	66.7%	67.2%	40.0%	78.8%	53.8%	47.1%	2.9%	32.8%	26.1%	43.8%	52.4%
