# Supplementary material for: Ten-year retrospective data analysis reveals frequent respiratory co-infections in hospitalized patients in Augsburg
Source: iScience. 2024 May 28;27(6):110136. doi: 10.1016/j.isci.2024.110136 (PMC11223076; doi:10.1016/j.isci.2024.110136)
Supplement: Data S1. Excerpt of the R script for the data analysis [file mmc2.pdf]

```
#### install and load packages ####
#install.packages("janitor")
#install.packages("dplyr")
#install.packages("gtsummary")
#install.packages("circlize")
#install.packages("RColorBrewer")
#install.packages("incidence2")
#install.packages("nnet")

library(janitor)
library(dplyr)
library(gtsummary)
library(circlize)
library(RColorBrewer)
library(incidence2)
library(nnet)
library(readxl)
library(ggplot2)

#Define project file
###Project folder
File_pathway       <- "/Users/mehmetg./Desktop/Martin_Krammer_data"
###Extract figures here
Figure_pathway     <- "/Users/mehmetg./Desktop/Martin_Krammer_data/exported-Figures"
##Extract tables here
Templates_pathway  <- "/Users/Mehmetg./Desktop/Martin_Krammer_data/exported-Excel"
##go and print the main project folder
setwd(File_pathway)
print(getwd())

##define script file name
XLSX_FILE = "Pathogen_data_clear.xlsx"
XLSX_FILE

## Import masterfile
##read the data
data1        <- read_xlsx(XLSX_FILE, 1)       #

#### data formatting ####
data1 <- Pathogen_data_clear

data1$data_coll <- as.Date(data1$data_coll)
data1$data_ana <- as.Date(data1$data_ana)

data1$plz <- factor(data1$plz, levels=0:2, labels=c("Others", "86XXX", "85XXX"))
data1$age_strat <- factor(data1$age_strat, level=0:1, labels=c("0-17 years", "≥18 years"))
data1$sex <- factor(data1$sex, levels=0:1, labels=c("Male", "Female"))
data1$samp_loc<- factor(data1$samp_loc, levels=1:4, labels=c("Pharynx/nose/mouth", "Bronchi/alveoli/trachea", "Others", "Not specified"))
data1$coinf_2 <- factor(data1$coinf_2, levels=0:1, labels=c("No co-infection", "Co-infection"))
data1$coinf_3 <- factor(data1$coinf_3, levels=0:2, labels=c("All negative", "Mono-infection", "Co-infection"))
data1$coinf_5 <- factor(data1$coinf_5, levels=0:4, labels=c("All negative", "Mono-infection", "Dual-infection", "Tri-infection", "Quad+-Infection"))
data1$coinf_bac_vir <- factor(data1$coinf_bac_vir, levels=0:3, labels=c("No co-infection", "Interviral co-infection", "Interbacterial co-infection", "Viral-bacterial co-infection"))

data1$AVP <- factor(data1$AVP, levels=0:1, labels=c("Negative", "Positive"))
data1$BPP <- factor(data1$BPP, levels=0:1, labels=c("Negative", "Positive"))
data1$BPPP <- factor(data1$BPPP, levels=0:1, labels=c("Negative", "Positive"))
data1$CPP <- factor(data1$CPP, levels=0:1, labels=c("Negative", "Positive"))
data1$HIBP <- factor(data1$HIBP, levels=0:1, labels=c("Negative", "Positive"))
data1$HINP <- factor(data1$HINP, levels=0:1, labels=c("Negative", "Positive"))
data1$HMP <- factor(data1$HMP, levels=0:1, labels=c("Negative", "Positive"))
data1$HRVP <- factor(data1$HRVP, levels=0:1, labels=c("Negative", "Positive"))
data1$IAS <- factor(data1$IAS, levels=0:1, labels=c("Negative", "Positive"))
data1$IBS <- factor(data1$IBS, levels=0:1, labels=c("Negative", "Positive"))
data1$LPP <- factor(data1$LPP, levels=0:1, labels=c("Negative", "Positive"))
data1$MCP <- factor(data1$MCP, levels=0:1, labels=c("Negative", "Positive"))
data1$MPP <- factor(data1$MPP, levels=0:1, labels=c("Negative", "Positive"))
data1$PAP <- factor(data1$PAP, levels=0:1, labels=c("Negative", "Positive"))
```

```
data1$RSP <- factor(data1$RSP, levels=0:1, labels=c("Negative", "Positive"))
data1$SPP <- factor(data1$SPP, levels=0:1, labels=c("Negative", "Positive"))


summary(data1)

#### Descriptive analyses of entire data set (n=16,520) after exclusion of fully non-interpretable tests (n=11) (Table 1) ####

#Age-stratified analysis for SEX
sex_by_agestrat <- data1 %>%
  tabyl(sex, age_strat, show_na = FALSE)
sex_by_agestrat
chisq.test(sex_by_agestrat)

#Age-stratified analysis for POSTCAL CODE
plz_by_agestrat <- data1 %>%
  tabyl(plz, age_strat, show_na = FALSE)
plz_by_agestrat
chisq.test(plz_by_agestrat)

#Age-stratified analysis for SAMPLE LOCATION
location_by_agestrat <- data1 %>%
  tabyl(samp_loc, age_strat, show_na = FALSE)
location_by_agestrat
chisq.test(location_by_agestrat)

#Age-stratified analysis for INFECTION TYPE - 3 CATEGORIES
coinf3_by_agestrat <- data1 %>%
  tabyl(coinf_3, age_strat, show_na = FALSE)
coinf3_by_agestrat
chisq.test(coinf3_by_agestrat)

#Age-stratified analysis for INFECTION TYPE - 5 CATEGORIES
coinf5_by_agestrat <- data1 %>%
  tabyl(coinf_5, age_strat, show_na = FALSE)
coinf5_by_agestrat <- coinf5_by_agestrat[-c(1, 2),,drop=F]
coinf5_by_agestrat
chisq.test(coinf5_by_agestrat)

#Age-stratified analysis for BAC-VIR-COMBINATION
bacvir_by_agestrat <- data1 %>%
  tabyl(coinf_bac_vir, age_strat, show_na = FALSE)
bacvir_by_agestrat <- bacvir_by_agestrat[-c(1),,drop=F]
bacvir_by_agestrat
chisq.test(bacvir_by_agestrat)


#### Comparison of sex and sampling location between infection types ####

#comparison of sex and sample location among age groups

#Creating subsets (adults vs. children)
dat_adults <- data1 %>% filter(data1$age_strat == "≥18 years")
dat_child <- data1 %>% filter(data1$age_strat == "0-17 years")

# comparison among children (Table S2)
dat_child %>%
  select(sex, coinf_3) %>%
  tbl_summary(by = coinf_3) %>%
  add_p()

dat_child %>%
  select(samp_loc, coinf_3) %>%
  tbl_summary(by = coinf_3)
fisher.test(as.factor(dat_child$samp_loc), as.factor(dat_child$coinf_3), simulate.p.value=TRUE)

# comparison among adults (Table S3)
dat_adults %>%
  select(sex, coinf_3) %>%
  tbl_summary(by = coinf_3) %>%
```

```
  add_p()

dat_adults %>%
  select(samp_loc, coinf_3) %>%
  tbl_summary(by = coinf_3) %>%
  add_p()

#Comparison of the proportion of patients with sample location ‘bronchi/alveoli/trachea’ between mono-infected and co-infected patients:
prop.test(x = c(45, 66), n = c(705, 4008)) #among children
prop.test(x = c(366, 852), n = c(508, 1451)) #among adults

#Testing of equal sex distribution among:
prop.test(x = c(9856, 6664), n = c(16520, 16520)) #entire sample

prop.test(x = c(2384, 1624), n = c(4008, 4008)) #mono-infected children
prop.test(x = c(405, 300), n = c(705, 705)) #co-infected children

prop.test(x = c(874, 577), n = c(1451, 1451)) #mono-infected adults
prop.test(x = c(338, 170), n = c(508, 508)) #co-infected adults


#### Comparing odds of co-infection between age groups by adjustment for test frequencies ####

#log. regression (co-infection (yes/no) dependent on age group (child/adult)), unadjusted
log_model_1 <- glm(formula = coinf_2 ~age_strat, family = binomial(), data = data1)
summary(log_model_1)
exp(0.36377) #odds of getting a co-infection when being an adult increases by 44% compared to being a child (odds = 1.44), p<0.001

#log. regression (co-infection (yes/no) dependent on age group (child/adult)), adjusted for number of pathogens tested for per patient
log_model_2 <- glm(formula = coinf_2 ~age_strat + numb_test, family = binomial(), data = data1)
summary(log_model_2)

#results interpretation
exp(-1.26460) #odds of getting a co-infection when being an adult decreases by 72% compared to being a child (odds = 0.28), p<0.001
exp(0.27056) #with every additional pathogen test the odds of detecting a co-infection increases by 31% (odds = 1.31), p<0.001


#Odds of presenting with more than 2 pathogens (tri/quad+-infections; supplementary analysis to the above Pearson's Chi squared test)
#ensure correct data level order for regression analysis
data1$age_strat <- relevel(data1$age_strat, ref = "≥18 years")
data1$coinf_3 <- relevel(data1$coinf_3, ref = "All negative")
data1$coinf_5 <- relevel(data1$coinf_5, ref = "All negative")
data1$samp_loc<- relevel(data1$samp_loc, ref = "Pharynx/nose/mouth")
coinf_bac_vir <- relevel(data1$coinf_bac_vir, ref = "No co-infection")

#create regression model
multinom.fit <- multinom(coinf_5 ~ age_strat + numb_test + sex + samp_loc, data = data1)
summary(multinom.fit) #provide summary of model outcomes
exp(coef(multinom.fit)) #give odds for all coefficients

#p-values of regression coefficients
z <- summary(multinom.fit)$coefficients/summary(multinom.fit)$standard.errors
p <- (1 - pnorm(abs(z), 0, 1)) * 2
p

#Supportive finding: Odds of detecting a tri- or quad+-infections compared to no co-infection was much higher among children (p<0.001)

#Odds of detecting various types of co-infections
multinom.fit <- multinom(coinf_bac_vir ~ age_strat + numb_bac_test + numb_vir_test + sex + samp_loc, data = data1)
summary(multinom.fit) #provide summary of model outcomes
exp(coef(multinom.fit)) #give Odds for all coefficients

#Supportive finding: Odds of a viral-bacterial co-infection were much higher for children than adults, followed by interviral co-infections and interbacterial co-infections (p<0.001 each)


#### Calculating cumulative incidences (exemplary for Figure S3) ####
```

```
dat1mo <- data1 %>%
  filter(data1$coinf_3 == "Mono-infection" & data1$data_coll >= "2013-01-01" & data1$data_coll <= "2017-12-31")
dat1co <- data1 %>%
  filter(data1$coinf_3 == "Co-infection" & data1$data_coll >= "2013-01-01" & data1$data_coll <= "2017-12-31")

#Calculating monthly cumulative incidences
inci_mo <- incidence(dat1mo, data_coll, interval = "month", groups = c(samp_loc, age_strat))
inci_co <- incidence(dat1co, data_coll, interval = "month", groups = c(samp_loc, age_strat))

#Plotting monthly incidences by age group with indication of sample location
facet_plot(inci_mo, facets = age_strat, fill = samp_loc, nrow = 1, n_breaks = 6, color = "white", date_format = "%Y", centre_dates =
FALSE, show_cases = FALSE, legend = c("bottom"), ylab = "Monthly incidence")
facet_plot(inci_co, facets = age_strat, fill = samp_loc, nrow = 1, n_breaks = 6, color = "white", date_format = "%Y", centre_dates =
FALSE, show_cases = FALSE, legend = c("bottom"), ylab = "Monthly incidence")

#Save plots as vector graphic
ggsave(file="incidence_plot.svg", plot=last_plot(), device = NULL,
       path = NULL,
       scale = 1,
       width = NA,
       height = NA,
       units = c("in", "cm", "mm", "px"),
       dpi = 300,
       limitsize = TRUE,
       bg = NULL)


##### Chord diagram #####

#loading and formatting data

##define script file name
Circle_all = "Co-Infection Circle Plot data.xlsx"
Circle_adults = "Co-Infection Circle Plot data _adults.xlsx"
Circle_children = "Co-Infection Circle Plot data _children.xlsx"


## Import masterfile
##read the data
circdat1        <- read_xlsx(Circle_adults, 1)       #


######
circdat1$Path_1 <- factor(circdat1$Path_1, levels=1:16, labels=c("B. pertussis",
                                                     "B. parapertussis",
                                                     "Chl. pneumoniae",
                                                     "H. influenzae type b",
                                                     "H. influenzae",
                                                     "L. pneumophila",
                                                     "M. catarrhalis",
                                                     "M. pneumoniae",
                                                     "S. pneumoniae",
                                                     "Adenovirus",
                                                     "Metapneumovirus",
                                                     "Rhinovirus",
                                                     "Influenza A",
                                                     "Influenza B",
                                                     "Parainfluenza 1, 2, 3",
                                                     "RSV"))

circdat1$Path_2 <- factor(circdat1$Path_2, levels=1:16, labels=c("B. pertussis",
                                                     "B. parapertussis",
                                                     "Chl. pneumoniae",
                                                     "H. influenzae type b",
                                                     "H. influenzae",
                                                     "L. pneumophila",
                                                     "M. catarrhalis",
                                                     "M. pneumoniae",
                                                     "S. pneumoniae",
```

```
                                                    "Adenovirus",
                                                    "Metapneumovirus",
                                                    "Rhinovirus",
                                                    "Influenza A",
                                                    "Influenza B",
                                                    "Parainfluenza 1, 2, 3",
                                                    "RSV"))


#Create chord diagram
circos.par(gap.after = c("B. pertussis" = 1, "B. parapertussis" = 1, "Chl. pneumoniae" = 1, "H. influenzae type b" = 1, "H. influenzae" =
1,
                    "L. pneumophila" = 1, "M. catarrhalis" = 1, "M. pneumoniae" = 1, "S. pneumoniae" = 15,
                    "Adenovirus" = 1, "Metapneumovirus" = 1, "Rhinovirus" = 1, "Influenza A" = 1, "Influenza B" = 1, "Parainfluenza 1, 2,
3" = 1, "RSV" = 15))

my_col <- c("B. pertussis"="midnightblue",
      "B. parapertussis"="midnightblue",
      "Chl. pneumoniae"="midnightblue",
      "H. influenzae type b"="midnightblue",
      "H. influenzae"="midnightblue",
      "L. pneumophila"="midnightblue",
      "M. catarrhalis"="midnightblue",
      "M. pneumoniae"="midnightblue",
      "S. pneumoniae"="midnightblue",
      "Adenovirus"="orange",
      "Metapneumovirus"="orange",
      "Rhinovirus"="orange",
      "Influenza A"="orange",
      "Influenza B"="orange",
      "Parainfluenza 1, 2, 3"="orange",
      "RSV"="orange")


######
my_col_linker_children <-c("Black", "Black", "Black", "#90A7C2", "Black", "#90A7C2", "Black", "#90A7C2", "Black", "Black", “Grey",
"Black", "Black", "Black", "Black", "Black", "Black", "Black", "Black", "Black", "Black", "Black", "Black", "Black", "Black", "Black",
"Black", "Black", "Black", "#90A7C2", "Black", "Black", "Black", "Black", "Black", "Black", "Black", "Black", "Black", "Black", "Black",
"#90A7C2", "Black", "#90A7C2", "Black", "#90A7C2", "Black", "Black", "Grey", "Black", "Black", "Black", "Black", "Black", "#90A7C2",
"#90A7C2", "#90A7C2", "Grey", "Grey", "Grey", "Grey", "Grey", "Grey", "Grey", "Black", "Black", "Black", "Black", "Black", "Black",
"Black", "Black", "Black", "Black", "Black", "#90A7C2", "Grey", "Grey", "Grey", "Grey", "Grey", "Grey", "Grey", "#90A7C2", "Black",
"Black", "Black", "Black", "Black", "Black", "Black", "Grey", "Grey", "Grey", "Grey", "Grey", "Grey", "Grey", "Black", "Orange", "Black",
"Black", "Black", "Black", "Black", "Black", "Black", "Black", "Black", "Black", "Black", "Black", "Orange", "Black", "Black", "Black",
"Black", "Black", "Black")


my_col_linker_adult <-c("Black", "Black", "Black", "Black", "Black", "Black", "Black", Black", "Black", "Black", "Black", "Black", “Black",
"Black", "Black", "Black", "Black", "Black", "Black", "Black", "Black", "Black", "Black", "Black", "Black", "Black", "Black", “Black", "Black",
"Black", "Black", "Black", "Black", "Black", "Black", "Black", "Black", "Black", "Black", "Black", "Black", "Black", “#90A7C2", "Black",
"Black", "Black", "Black", "Black", "Black", "Black", "Black", "Black", "Black", "Black", "Black", "#90A7C2", “Black", "#90A7C2", "Black",
"Black", "Grey", "Grey", "Black", "Black", "Black", "Black", "Black", "Black", "Black", "Black", "Black", “Black", "Black", "Black", "Black",
"Black", "#90A7C2", "Black", "Black", "Black", "Black", "Black", "Black", "Black", "Black", "Black", “Black", "Black", "Black", "Black",
"Black", "Black", "Black", "Black", "Grey", "Grey", "Black", "Black", "Black", "Black", "Black", "Black", "Black", "Black", "Black", "Black",
"Black", "Black", "Black", "Black", "Black", "Black", "Black", "Black", "Black", "Black",
"Black", "Black", "Black", "Black" )

my_col_linker_all <- c("Black", "Black", "Black", "#90A7C2", "Black", "Black", "Black", "#90A7C2", "Black", "Black", "Black",
 "Black", "Black", "Black", "Black", "Black", "Black", "Black", "Black", "Black", "Black", "Black", "Black", "Black", "Black", "Black", "Black",
"Black", "Black", "Black", "Black", "Black", "Black", "Black", "Black", "Black", "Black", "Black", "Black", "Black", "Black", "Black",
"#90A7C2", "Black", "Black", "Black", "#90A7C2", "Black", "Black", "Black", "Black", "Black", "Black", "Black", "Black", "#90A7C2",
"Black", "#90A7C2", "Black", "Black", "Grey", "Grey", "Black", "Black", "Grey", "Black", "Black", "Black", "Black", "Black", "Black",
"Black", "Black", "Black", "Black", "Black", "#90A7C2", "Black", "Black", "Grey", "Black", "Black", "Black", "Black",
 "Black", "Black", "Black", "Black", "Black", "Black", "Black", "Black", "Black", "Black", "Grey", "Grey", "Black", "Black", "Grey", "Black",
"Orange", "Black", "Black", "Black", "Black", "Black", "Black", "Black", "Black", "Black", "Black", "Black", "Black", "Black", "Black",
"Black", "Black", "Black", "Black", "Black")


#####
########Save plots as vector graphic
svg("Figure 2b.svg",
```

```
    width = 12, height = 9)
chordDiagram(circdat1, transparency = 0.4, grid.col = my_col,
             #col = colorRamp2(seq(0, 1, 0.2), brewer.pal(6, "Blues")),
             col = my_col_linker_adult,
             order = c("B. pertussis",
                       "B. parapertussis",
                       "Chl. pneumoniae",
                       "H. influenzae type b",
                       "H. influenzae",
                       "L. pneumophila",
                       "M. catarrhalis",
                       "M. pneumoniae",
                       "S. pneumoniae",
                       "Adenovirus",
                       "Metapneumovirus",
                       "Rhinovirus",
                       "Influenza A",
                       "Influenza B",
                       "Parainfluenza 1, 2, 3",
                       "RSV"),
             link.sort = TRUE,
             link.largest.ontop = TRUE,
             symmetric = TRUE,
             link.visible = circdat1[[3]] >= 50,
             link.decreasing = FALSE,
             annotationTrack = c("grid"),
             annotationTrackHeight = c(0.03, 0.3),
             preAllocateTracks = list(track.height = max(strwidth(unlist(dimnames(circdat1))))))

circos.track(track.index = 1 ,panel.fun = function(x, y) {
  circos.text(CELL_META$xcenter, CELL_META$ylim[1], CELL_META$sector.index,
              facing = "clockwise", niceFacing = TRUE, adj = c(0, 0.5)) }, bg.border = NA)

legend(0.8,-0.7, legend=c("Viruses", "Bacteria"), col=c("orange","midnightblue"),box.lty=0, pch=15, pt.cex=2, cex=1)
dev.off()
circos.clear()


#### 95% confidence intervals of pathogen co-infection rates (based on 2008-2017 data) ####

#CIs for children (0-17 years)
prop.test(29,53,correct=FALSE)
prop.test(3,5,correct=FALSE)
prop.test(5,5,correct=FALSE)
prop.test(14,14,correct=FALSE)
prop.test(266,335,correct=FALSE)
prop.test(0,1,correct=FALSE)
prop.test(115,141,correct=FALSE)
prop.test(8,9,correct=FALSE)
prop.test(258,316,correct=FALSE)
prop.test(154,619,correct=FALSE)
prop.test(68,311,correct=FALSE)
prop.test(206,997,correct=FALSE)
prop.test(116,560,correct=FALSE)
prop.test(34,201,correct=FALSE)
prop.test(61,366,correct=FALSE)
prop.test(160,1321,correct=FALSE)

#CIs for adults (≥18 years)
prop.test(4,7,correct=FALSE)
prop.test(0,0,correct=FALSE)
prop.test(2,5,correct=FALSE)
prop.test(20,38,correct=FALSE)
prop.test(366,647,correct=FALSE)
prop.test(6,43,correct=FALSE)
prop.test(49,73,correct=FALSE)
prop.test(5,17,correct=FALSE)
prop.test(355,572,correct=FALSE)
prop.test(11,24,correct=FALSE)
prop.test(12,49,correct=FALSE)
```

```
prop.test(65,197,correct=FALSE)
prop.test(52,297,correct=FALSE)
prop.test(10,44,correct=FALSE)
prop.test(16,82,correct=FALSE)
prop.test(18,78,correct=FALSE)


#### Exact binomial tests as part of Proportional Distribution Model (exemplary for co-infection pairings of B. pertussis) ###
#comparison with B. parapertussis
binom.test(1, 804, 0.0470444632669354/804, alternative = "two.sided", conf.level = 0.95)

#comparison with Chl. pneumoniae
binom.test(0, 618, 0.0688432605746701/618, alternative = "two.sided", conf.level = 0.95)

#comparison with H. influenzae type b
binom.test(0, 618, 0.0601522368114556/618, alternative = "two.sided", conf.level = 0.95)

#comparison with H. influenzae
binom.test(21, 652, 2.9128131261511/652, alternative = "two.sided", conf.level = 0.95)

#comparison with L. pneumophila
binom.test(0, 616, 0.011519364448858/616, alternative = "two.sided", conf.level = 0.95)

#comparison with M. catarrhalis
binom.test(5, 640, 0.946280343757912/640, alternative = "two.sided", conf.level = 0.95)

#comparison with M. pneumoniae
binom.test(0, 619, 0.0571565104029033/619, alternative = "two.sided", conf.level = 0.95)

#comparison with S. pneumoniae
binom.test(21, 650, 3.10771732872799/650, alternative = "two.sided", conf.level = 0.95)

#comparison with adenovirus
binom.test(0, 297, 4.0185124788273/297, alternative = "two.sided", conf.level = 0.95)

#comparison with human metapneumovirus
binom.test(0, 297, 1.87597122229465/297, alternative = "two.sided", conf.level = 0.95)

#comparison with human rhinovirus
binom.test(3, 270, 6.24408914305251/270, alternative = "two.sided", conf.level = 0.95)

#comparison with influenza A virus
binom.test(0, 297, 2.37676367981103/297, alternative = "two.sided", conf.level = 0.95)

#comparison with influenza B virus
binom.test(0, 297, 0.521223164396152/297, alternative = "two.sided", conf.level = 0.95)

#comparison with parainfluenza virus 1, 2, 3
binom.test(0, 297, 2.38071122195773/297, alternative = "two.sided", conf.level = 0.95)

#comparison with RSV
binom.test(0, 297, 4.37120245551881/297, alternative = "two.sided", conf.level = 0.95)


#### Additional analysis: Age distribution stratified by age group and infection type ####

#load and install additionally required packages
install.packages("viridis")
install.packages("ggplot2")
install.packages("hrbrthemes")
library(viridis)
library(ggplot2)
library(hrbrthemes)


#Plotting histograms by test results - children
  #Histograms for children
  data1 %>%
    filter(age_strat == "0-17 years") %>%
    ggplot(aes(x = age, color=coinf_3, fill=coinf_3)) +
```

```
  geom_histogram(alpha=0.6, binwidth = 1) +
  scale_fill_viridis(discrete=TRUE) +
  scale_color_viridis(discrete=TRUE) +
  theme_ipsum() +
  theme(
    legend.position="none",
    panel.spacing = unit(0.1, "lines"),
    strip.text.x = element_text(size = 8)) +
  facet_grid(vars(coinf_3),scales = "free")

 #Histograms for adults
 data1 %>%
  filter(age_strat == "≥18 years") %>%
  ggplot(aes(x = age, color=coinf_3, fill=coinf_3)) +
  geom_histogram(alpha=0.6, binwidth = 1) +
  scale_fill_viridis(discrete=TRUE) +
  scale_color_viridis(discrete=TRUE) +
  theme_ipsum() +
  theme(
    legend.position="none",
    panel.spacing = unit(0.1, "lines"),
    strip.text.x = element_text(size = 8)) +
  facet_grid(vars(coinf_3),scales = "free")


#Save as svg file
ggsave(file="histogram_strat_age.svg", plot=last_plot(), device = NULL,
       path = NULL,
       scale = 1,
       width = NA,
       height = NA,
       units = c("in", "cm", "mm", "px"),
       dpi = 300,
       limitsize = TRUE,
       bg = NULL)
```
